# Complete Genome Sequence of a Reassortant H14N2 Avian Influenza Virus from California

**DOI:** 10.1128/genomeA.00543-13

**Published:** 2013-08-01

**Authors:** Walter M. Boyce, Seth Schobel, Vivien G. Dugan, Rebecca Halpin, Xudong Lin, David E. Wentworth, LeAnn L. Lindsay, Eva Mertens, Magdalena Plancarte

**Affiliations:** Department of Pathology, Microbiology and Immunology, School of Veterinary Medicine, University of California, Davis, California, USAa; J. Craig Venter Institute, Rockville, Maryland, USAb

## Abstract

We report the complete genome sequence of a reassortant H14N2 avian influenza virus isolated in 2011 from a northern shoveler in California. This introduced Eurasian subtype acquired seven segments from North American viruses and circulated in the Pacific Flyway 1 year after its detection in the Mississippi Flyway.

## GENOME ANNOUNCEMENT

Prior to 2010, the only known avian influenza viruses (AIV) belonging to the H14 subtype were sequences from mallards (*Anas platyrhynchos*) and a herring gull (*Larus argentatus*) sampled at the Caspian Sea in 1982 (1). In 2010, H14N6 and H14N8 viruses were isolated from sea ducks (*Clangula hyemalis* [*n* = 2] and *Melanitta fusca* [*n* = 1]) in the U.S. Mississippi Flyway, demonstrating the first known movement of H14 into the Western hemisphere (2, 3).

We isolated an H14N2 virus from the cloacal swab of a Northern shoveler (*Anas clypeata*) sampled on 29 October 2011 in the Suisun Marsh near San Francisco Bay, CA. This virus, A/northern shoveler/California/2696/2011(H14N2), was compared to all H14 sequences available on GenBank (*n* = 13).

The HA gene of A/northern shoveler/California/2696/2011(H14N2) shares 97% nucleotide identity with six H14 viruses isolated in North American in 2010 and 89% identity with the seven H14 viruses isolated in Central Asia in 1982. Although the H14 subtype can support a highly pathogenic phenotype (4), amino acids at the HA cleavage site (PDKQTK) were identical to the 2010 H14 isolates and lacked the polybasic amino acid signature characteristic of a highly pathogenic virus. In common with other North American isolates, the HA gene contained phenotypic markers of increased binding to α-2,6 sialic acid receptors (I151T and V210I) and increased virulence in mammals (A263T).

The sudden emergence in 2010 of H14 viruses in sea ducks and dabbling ducks in North America was unprecedented. Some of the 2010 isolates contained NA and NS genes of Eurasian origin, and all six isolates were obtained from the same region, indicating a recent introduction into the Mississippi Flyway. Analysis of A/northern shoveler/California/2696/2011(H14N2) revealed extensive reassortment of all segments other than HA (NA, PA, NS, PB1, PB2, M, and NP), sharing 99% identity with contemporary North American isolates circulating in both the Pacific and Mississippi Flyways.

The isolation of A/northern shoveler/California/2696/2011(H14N2) supports the hypotheses that H14 viruses circulated in North America from 2010 to 2011 and that mixed infections readily produced reassortant viruses in different flyways separated by 2000 km within a single year (5, 6).

### Nucleotide sequence accession numbers. 

The GenBank accession numbers for the H14N2 complete genome sequence are CY146897 through CY146904.
